# Clinical image: spondylodiscitis as a complication of urosepsis caused by extracorporeal shock wave lithotripsy for kidney stones

**DOI:** 10.1093/omcr/omac144

**Published:** 2023-01-18

**Authors:** Pasquale Gallina, Maddalena Dardo, Agnese Pedone, Fabrizio Travaglini

**Affiliations:** Neurosurgery Unit, Department of Neurosciences, Psychology, Drug Research and Child Health, University of Florence, Florence, Italy; Neurosurgery Unit, Department of Neurosciences, Psychology, Drug Research and Child Health, University of Florence, Florence, Italy; Neurosurgery Unit, Department of Neurosciences, Psychology, Drug Research and Child Health, University of Florence, Florence, Italy; Urology Unit, Careggi University Hospital, Florence, Florence, Italy

Extracorporeal shock wave lithotripsy (ESWL), a well-established treatment for urinary stones [1], is not free of complications [2]. This is the first report of spondylodiscitis following ESWL.

**Figure 1:**
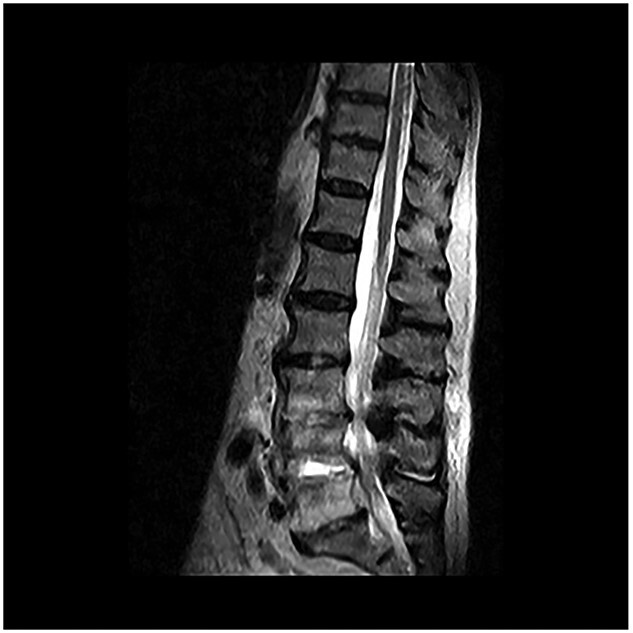
Sagittal T2-weighted magnetic resonance imaging obtained 2 weeks after extracorporeal shock wave lithotripsy for kidney stones complicated by *Escherichia coli* septicemia. Image shows increased signal intensity of L3–L4 and L4–L5 discs and adjacent vertebral bodies. The features indicate ongoing spondylodiscitis.

A woman in her 70s had right renal colic for 3 months. On 9 December 2020, she consulted a urologist at a regional hospital. Clinical examination showed negative results, while abdominal ultrasound revealed a 16-mm right pyelic stone. Urinalysis performed on 12 December 2020 revealed leukocyturia and microhematuria. On 16 February 2021, the consentient patient underwent ESWL. The early post-operative course was uneventful. Thirty-six hours after the procedure, she experienced a fever with rigor and chills and progressive onset of lumbar pain. Home therapy with oral cefixime was unsuccessful, and she was re-admitted to the hospital 7 days after ESWL (23 February 2021). Increased white blood cells (11 600/μl), serum procalcitonin (19.84 ng/ml) and C-reactive protein (14.72 mg/dl) were detected. Hemoculture was positive for extended-spectrum beta-lactamases producing *Escherichia coli*, resistant to beta-lactamine and ciprofloxacin. Abdominal computerized tomography showed the unmodified stone. Hydronephrosis was not detected. Intravenous perfusion of gentamycin and co-trimoxazole allowed resolution of the sepsis in 5 days. The patient continued to take oral co-trimoxazole for a week after hospital discharge. In the meantime, the lumbar pain increased in intensity, which led her to consult a neurosurgeon at our university hospital. Magnetic resonance imaging (23 February 2021) showed spondylodiscitis at levels L3, L4, L5 (Fig. 1), which was treated with a cycle of parenteral antibiotics according to the modality described above. The patient was also fitted with an orthopedic brace, which gave complete relief of pain in 1 month. She was made aware of the possible occurrence over time of clinical and imaging features of lumbar stenosis as a consequence of vertebral and discs post-spondylodiscitis deformity. Weight loss and swimming were recommended. Eighteen months after ESWL, the patient was asymptomatic and presented only a modest limitation of lumbar spine motility. Regarding the stone, the patient, free from colic, refused to undergo retrograde intrarenal surgery.

Spondylodiscitis in the course of urinary infections is a consequence of hematogenous spread of infection [3]. A secondary spondylodiscitis related to *Enterobacter cloacae* septicemiamanifesting after ESWL for urethral stone was observed by Kamanli *et al*. [4]. However, in that case, urinary tract infection and obstructive uropathy caused by stones were pre-existent to ESWL [4]. Even the spondylodiscitis was probably pre-existent, so that its nexus with ESWL is completely uncertain [4]. Differently from the observation of Kamanli *et al*. [4], in our patient, the absence of obstruction of the urinary tract (possible *pavulum* of bacteria), and of urosepsis, implied that septicemia was a consequence of the bacterial spread triggered by ESWL. However, a latent pre-ESWL urinary tract infection cannot be excluded on the basis of the previous leukocyturia.

Pre-ESWL urine culture is mandatory [5]. Spondylodiscitis after complicated ESWL is an extremely rare possibility. Early diagnosis/treatment may result in the prevention of epidural/subdural/intramedullary abscess and/or minimization of disc space narrowing and bony destruction [3], this limiting the risk of lumbar stenosis occurrence.
